# Risk of Soil-Transmitted Helminthiasis among Agrarian Communities of Kogi State, Nigeria

**DOI:** 10.5334/aogh.2563

**Published:** 2019-09-11

**Authors:** Joy T. Anunobi, Ikem C. Okoye, Ifeanyi O. Aguzie, Yvonne E. Ndukwe, Onyekachi J. Okpasuo

**Affiliations:** 1 Science Laboratory Technology Department, Federal Polytechnic, Idah, Kogi State, NG; 2 Parasitology and Public Health Unit, Department of Zoology and Environmental Biology, University of Nigeria, Nsukka, Enugu State, NG

## Abstract

**Background:**

Soil-transmitted helminths (STH) have remained a major threat to humans, especially children in developing countries, including Nigeria. Interventions have always been geared towards school-aged children, neglecting preschool-aged children and occupational risk adults. The Soil-Transmitted Helminthiasis Advisory Committee (STHAC) recently suggested incorporating other at-risk groups.

**Objective:**

This study assessed the associated risk of STH infection among agrarian communities of Kogi State, Nigeria.

**Methods:**

A total of 310 individuals of all ages participated in the cross-sectional survey. Stool samples were analyzed using standard Kato-Katz method.

**Results:**

A total of 106 (34.2%) individuals were infected with at least one STH. Hookworm was the most prevalent (18.1%); followed by *Ascaris lumbricoides* (16.8%). Worm intensity was generally light. Prevalence of infection was similar between four age groups considered (preschool, school, ‘women of reproductive age’ and older at-risk group). Poor socio-economic status (SES) was a major risk for STH infection. Using a 20-asset based criteria, 68 (23.1%) and 73 (24.7%) of 295 questionnaire respondents were classified into first (poorest) and fifth (richest) wealth quintiles respectively. Risk of infection with STH was 60% significantly lower in the richest wealth quintile compared to the poorest (Prevalence Ratio [PR] = 0.4843, 95% CI = 0.2704–0.8678, p = 0.015). Open defecators were more likely to harbour STH than those who did not (PR = 1.7878, 95% CI = 1.236–2.5846, p = 0.00201). Pit latrine and water closet toilets each approximately reduced STH infection by 50% (p < 0.05).

**Conclusion:**

Preventive chemotherapy for all age groups, health education and provision of basic amenities especially toilets are needed in order to achieve the goal toward the 2020 target of STH control.

## Introduction

Soil-transmitted helminths (STH) are intestinal worms whose immature stages require a period of incubation in the soil before becoming infective [1]. The most common are *Ascaris lumbricoides, Trichuris trichiura*, the hookworms (*Ancylostoma duodonale* and *Necator americanus*) and *Strongyloides stercoralis* [2]. They are transmitted by eggs present in infected human faeces, which contaminate the soil in areas where sanitation is poor. STH infections are included in the list of the world’s neglected tropical diseases, NTDs, and are the most common infections among the poorest and most deprived communities [2,3]. Transmission of *A. lumbricoides* and *T. trichiura* is primarily through oral-faecal route (usually by ingestion of parasite eggs in faeces), whereas hookworm species and *S. stercoralis* are through active skin penetration of infective larva.

STH infections are among the most common chronic human infections in most regions of the world; with approximately 1.5 billion people infected globally [2]. It accounts for a global burden of 3.3 million of disability-adjusted life years [4]. Approximately 270 million preschool-aged children (PSAC) and 600 million school-age children (SAC) are at risk of infection. They live in areas where transmission of these parasites are intense and in need of treatment and preventive interventions [2,5].

Intestinal helminthiasis prevalence in Nigeria has remained unchanged since the 1970s [6]. Nigeria has the highest burden and endemicity [3,7]. A larger fraction of those affected are young children of ages 5 to 14 years, living in rural areas and urban slums [6,7,8,9]. The major contributors to persistence of infections are cultural, socio-economic and environmental factors [6,7]. Unhygienic and common practice of indiscriminate defecation or dumping excrement have persisted in Nigeria [7,10,11]. There has been little success in the introduction of latrines to rural Nigeria [12]. A temporal data available from 1990 through 2015 indicate a marginal decrease in the use of improved sanitation facilities from 38% to 29%, and small increase in open defecation from 24% to 25% [13]. Currently, Nigeria is ranked as one of the nations in the world with the highest number of people practicing open defecation, estimated at over 46 million people [14].

In Nigeria, there are still states where there are limited to no epidemiological information on STH infections; Kogi State is one of those with limited information. Majority of the communities in Ibaji and Igalamela-Odolu Local Government Areas (LGAs) of Kogi State are farming communities where children and adults are fully exposed to risks of helminth infection. In agrarian communities, practices such as walking on bare feet, open defecation, eating of fallen fruits and raw unwashed vegetables with unwashed hands in farmlands could predispose individuals to STH infections [9,15]. Majority of the agrarian communities are warm and moist for most of the year, creating a good environment for the parasites to develop all year round.

Therefore the aim of the present study was to ascertain the associated risk factors of STHs among the agrarian communities of Ibaji and Igalamela-Odolu LGAs. Specifically the objectives were to assess, the prevalence and intensity of soil-transmitted helminthiasis; proportion of individuals with low, moderate and high worm intensity; age-related distribution of STHs infection; socio-economic status as risk for STHs infection; and risks associated with knowledge, attitude and practices (KAP). These objectives were pursued in view of the year 2016 recommendations of The Soil-Transmitted Helminthiasis Advisory Committee (STHAC) in relation to the revision of the 2012 Strategic Plan for STH control [16,17]. The prevalence and intensity of soil-transmitted helminthiasis in the LGAs were assessed in view of WHO 2020 milestones number 3 that all countries requiring PC for STH have less than 1% prevalence of moderate to high intensity [16,17]. Though this study was localized to agrarian communities of Kogi State as arbitrary ‘sentinels sites’ (milestone number 2). Kogi State was included in the recent epidemiological mapping of schistosomiasis and soil-transmitted helminthiasis by the Federal Government of Nigeria [18]. However, the mapping was only school based, leaving out vulnerable adults. The WHO 2012 Strategic Plan for STH control recommends preventive chemotherapy for SAC and PSAC, and has now included deworming for women of reproductive age. STHAC and some authors have suggested inclusion of other at risk groups [17]. The choice to use the agrarian community was due to the persistent risk of infection in such settings, which may justify need for localized mass drug administration (MDA) strategies. Though current WHO STH control strategy depends majorly on preventive chemotherapy (PC), the role of socio-economic status and KAP cannot be ignored. The authors believed that the importance of water, sanitation and hygiene (WASH) in control of STH could be assessed in relation to socio-economic status and KAP. Therefore, socio-economic status of the study population was assessed using an asset-based approach, which involved classification of individuals into wealth quintiles in relation to possession of arbitrary but systematically chosen assets.

## Methods

### Study design

The study employed a household-based, explorative, cross-sectional study design.

### Study area

The study area comprises Ibaji and Igalamela-Odolu LGAs of Kogi State, Nigeria. In Ibaji LGA, six communities were sampled. They are Ejule-Ojebe, Onyedega, Aneke, Inyano, Ichalla-Ajode and Otiaka. Five communities were sampled in Igalamela-Odolu LGA. They are Ogbagba, Okpachala, Ofuloko, Ogbogbo and Aikpele.

Ibaji LGA is located on the Eastern flank of Kogi State. The north easterly line of equal latitude and longitude passes through the LGA. Ibaji LGA has an area of 1,377 km^2^ and a population of 128,129 [19]. Geographically, it is located between longitudes 6°45′E and 7°00′E and latitudes 6°45′ and 7°00′N. Its inhabitants are primarily Igala speaking tribe [20]. High volume of rainfall and warm temperature result in lush vegetation and good conditions for cultivation of food crops such as rice, vegetables, yam, cassava, sorghum, maize, millet, cowpea, and groundnut which predominate the agricultural practice in the LGA. The major occupation is crop farming with some of them taking to fishing activities to supplement their meals and earn income for themselves. Only few of them are civil servants. The area suffers from poor infrastructural facilities such as absence of good road networks, electricity, pipe-borne water, health centres and communication network; and houses in these communities are mainly made of mud walls and thatched roofs [21].

Igalamela-Odolu LGA has its headquarters in the town of Ajaka (7°10′16″N and 6°49′35″E). The northeasterly line of equal latitude and longitude passes through the LGA. It has an area of 2,175 km² and a population of 148,020 [19]. Majority of the communities in the LGA are made up of crop farmers while few others are civil servants. Basic amenities such as pipe-borne water, electricity, toilets and accessible roads are lacking in most rural communities in the LGA.

### Study Participants and study size

Communities sampled were selected randomly. The study population consisted of both male and female adults and children who are resident in the selected communities.

Sample size was estimated using the sample size estimation formula by Yamane [22].

{\rm{n}}\ = \ \frac{{\rm{N}}}{{{\rm{(1 + N}}{{\rm{e}}^{\rm{2}}}{\rm{)}}}}

Where, n = sample size, N = total number of study population (based on 2006 census). e = probability level (P = 0.05). The estimated sample size was 399. Though 310 people consented to the study.

Within each chosen community, selection of the first households was by a systematic random sampling. Thereafter alternate households were enlisted. Within each enlisted household, all members were sampled to avoid the usual displeasure of those omitted [23]. Sample collections took place between February and April 2017, though the study duration was between August 2016 and May 2017.

Considering the additional cost incurred from additional drug provision to all age groups as has been recommended by STHAC and other authors, this study partitioned our study population into four age groups: PSAC (0–4 yrs.), SAC (5–18 yrs., though 14 years was not used as the upper limit), ‘women of reproductive age and men of same age bracket’ (19–45 yrs.), and older at risk group (>45 yrs.). This is to enable comparison of STHs infections among the age groups. It is believed that where prevalence of STH infection is similar between the age groups, it would support a need for inclusion of all age group in preventive chemotherapy interventions.

### Ethical Approval

The study protocol was approved by the Ethical Committee of the Kogi State Ministry of Health, Lokoja, Kogi State. The ethical approval was assigned the reference number MOH/KGS/1376/1/79.

### Advocacy and community sensitization

Prior to commencement of the study, advocacy visits were made to designated communities. The traditional rulers of each of the LGAs were visited and briefed on the study objectives. Written permissions were obtained from each of the traditional rulers indicating their consent on behalf of the entire communities. The community and household heads were also well-briefed on the objectives of the study. Verbal permissions were obtained from both the community heads and heads of each household visited. Each member of a household was given a consent form on which interest to participate in the study was indicated. Consent for children was obtained from their parents/care-givers.

### Stool Sample collection and processing

Fresh stool samples for helminth screening were collected from each of the subjects into a coded, dry, leak proof and sterilized sample container, each participant having been briefed to ensure that no urine, water, soil or other contaminants entered the container. The samples were received early in the morning, recorded appropriately and processed immediately. Samples which were not processed in the field were kept refrigerated using a portable cooler and later transported the same day to the laboratory for processing within a maximum of three hours.

Stool specimen was processed using the Kato-Katz technique and the Kato-Katz stool examination kit (Vestergaard Fradson, Switzerland) was used [24]. Stool specimens were processed within three hours of collection and examined microscopically within one hour of preparation to avoid over clearance of hookworm eggs [25]. The presence of STH parasites eggs in any of the stool samples were noted as positive. Microscopic examinations was repeated days after the slide preparation for the confirmation of the presence of *Trichuris trichiura* and *Ascaris lumbricoides* eggs as a quality control procedure.

### Assessment of demographic and socio-economic status

#### Use of questionnaires

A well-structured, pre-tested questionnaire was administered to each participant. The questionnaire was interview-based since most participants had no formal education and could not read nor write. However, those that could read and write provided the information at their convenience. Responses for 0–4-year-old participants comprising 18.6% (n = 55) of the study population were provided by their parents/guardians.

The questionnaire was sectioned into nine parts: (i.) basic demographic information, (ii.) perception and knowledge about intestinal helminths, (iii.) community and household characteristics, (iv.) hygiene practices, (v.) farm/home activities, (vi.) use of shoes or sandals, (vii.) use of health services, (viii.) history of deworming, and (ix.) possession of domestic animals.

### Visual inspection of compounds

Complementary information to questionnaire responses was obtained by visual inspections. Inspection of compounds was made and the sanitary condition and presence or absence of sewage disposal system noted. Wearing of shoes at home was assessed by visual observation. Water supply for drinking and washing of hands after using the toilets or urinals was noted. Children playing on sand were noted as well as those walking around the compound without shoes.

### Quantitative variables

Prevalence of infection was computed as proportion of individuals infected to total number examined and presented in percentages. Mean intensity was obtained based on number of eggs per gramme of faeces (epg) of the infected individual and categorized into light, moderate and heavy as established by WHO [26]. The number of eggs obtained was multiplied by 24 which is the standard for the Kato-Katz technique. Mean intensity was obtained as an average number of parasite eggs per infected individuals. Infection status and questionnaire variables were dummy-coded for statistical analysis purpose. All 310 participants responded to the questionnaire. Incomplete response led to exclusion of 15 (4.8%) questionnaires, leaving 295. Prevalence and infection intensity report on the 310 participants was provided while socio-economic status and risk estimates used the 295 properly completed questionnaires.

### Data analysis

Prevalence of infection was compared using chi-square analysis or Fisher’s exact test. Asset-based approach was used to group study participants into different socio-economic status [27,28]. Possession of different assets considered indicators of socio-economic status (SES) was used to develop a single composite variable SES. Twenty assets were used (S1 Table). Principal component analysis (PCA) extracted the key components. Seven principal components were extracted and the first component which explained 24.9% of total variation served as SES; its factor score loading helped group individuals into quintiles (S1 Table) [27]. Quintile 1 (Q1) represented the poorest who possessed assets associated with lowest extreme of SES, or who did not possess most assets associated with wealth. Quintile 2 (Q2) were poor, Q3 were average (neither poor nor rich), Q4 were rich while Q5 were the richest. The five quintiles were dummy-coded and used for further analysis. Some assets were either combined or excluded; possession of tiled floor was combined with cement floor and both retained as “cement floor” because only 8 persons used tiles. Possession of dog, pig and cow were removed from the PCA because of their scarcity in the population. Possession of these assets and other attributes were compared between the different SES by chi-square analysis.

Risk of infection associated with SES, knowledge and perception, attitude and practices are presented as the prevalence ratio (PR). Poisson regression model with robust variance was fitted to estimate the PR, 95% confidence interval and associated p-values [29]. Data was analyzed using RStudio and SPSS version 20.0 (IBM Corporation, Armonk, USA) [30]. Level of significance was set at 95% probability level.

## Results

### Prevalence and intensity of soil-transmitted helminthiasis in Ibaji and Igalamela-Odolu LGAs

**Figure 1:**
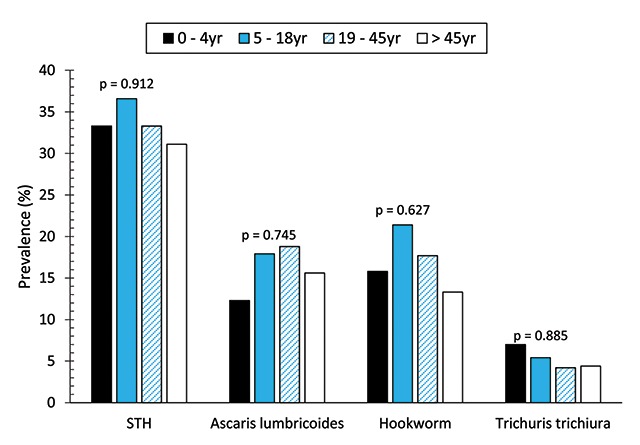
Prevalence of soil-transmitted helminths by age groups.

Prevalence of STH in Ibaji and Igalamela-Odolu LGAs was 34.2%, 106 out of 310 persons examined were infected with at least one of the STH, *Ascaris lumbricoides*, hookworm and *Trichuris trichiura*. Hookworm was the most prevalent (18.1%) and *T. trichiura* the least (5.2%) (Table 1). Difference in prevalence of the three STH was significant (χ^2^ = 27.097, p < 0.0001). STH infections were recorded in the two LGAs. Out of 167 and 143 participants, 58 (34.7%) and 48 (33.6%) respectively were infected in Ibaji and Igalamela-Odolu LGAs; the difference was not significant (χ^2^ = 0.046, p = 0.829). Prevalence of STH infection collectively and specifically was similar between the four age groups considered, namely PSAC (0–4 yr), SAC (5–18 yrs), ‘women of reproductive age and men in similar age bracket’ (19–45 yrs), and older at-risk groups (>45 yrs) (Figure 1).

**Table 1:** Overall prevalence of soil-transmitted helminths. (N = 310).

	No. Infected (%)	Egg count (epg)	

STH	106 (34.2)	**Intensity (Mean ± SD)**	**Min.–Max.**

*Ascaris lumbricoides*	52 (16.8)	216.92 ± 129.81	48–600
Hookworm	56 (18.1)	373.71 ± 318.10	48–2232
*Trichuris trichiura*	16 (5.2)	156.00 ± 143.73	48–576

N = number examined. Epg = egg per gram. SD = standard deviation.

**Figure 2:**
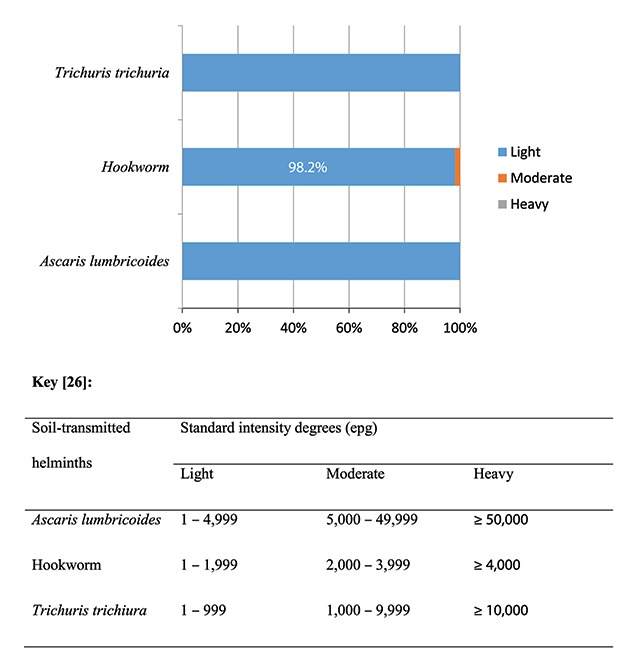
Intensity of soil transmitted helminths in agrarian communities of Ibaji and Igalamela-Odolu Local Government Area, Kogi State.

Mean egg counts are summarized in Table 1. In Figure 2 intensity of the three STH based on WHO standard categories is shown [26]. Intensities of the three helminths were largely light. Collectively, prevalence of moderate and heavy intensity of STH infection was <1%, though moderate intensity of infection only occurred for hookworm. Moderate intensity of hookworm infection only occurred in 1.8% of individuals infected.

### Demographics and socio-economic status of questionnaire respondents

All 310 participants responded to questionnaire. Incomplete response led to exclusion of 15 (4.8%) questionnaires, leaving 295. Demographics and SES of respondents are summarized in Tables 2 and 3. Majority of the respondents were students (38.6%), farmers (36.9%) and PSAC (21.4%) (Table 2). Majority of the participants lived in houses made of mud (50.5%) and cement (46.8%) floors (Table 3). Over 41% of respondents had electricity and 51.9% had pipe-borne water.

**Table 2:** Overall demographics of questionnaire respondents of agrarian communities in Ibaji and Igalamela-Odolu Local Government Areas, Kogi State (n = 295).

Characteristics	Frequency (%)

**LGA**	
Ibaji	154 (52.2)
Igalamela-Odolu	141 (47.8)
**Community**	
Onyedega	19 (6.4)
Ejule	15 (5.1)
Inyano	15 (5.1)
Aneke	15 (5.1)
Ichalla-Ajode	72 (24.4)
Otiaka	18 (6.1)
Ogbagba	14 (4.7)
Okpachala	28 (9.5)
Ofuloko	18 (6.1)
Ogbogbo	53 (18.0)
Aikpele	28 (9.5)
**Sex**	
Male	153 (51.9)
Female	142 (48.1)
**Age (year)**	
0–4	55 (18.6)
5–18	107 (36.3)
19–45	89 (30.2)
>45	44 (14.9)
**Marital Status**	
Single	173 (58.6)
Married	122 (41.4)
**Occupation**	
Pre-school	63 (21.4)
Student	114 (38.6)
Farming	109 (36.9)
Fishing	1 (0.3)
Civil Servant	6 (2.0)
Others	2 (0.7)

n = number of respondents.

**Table 3:** Socio-economic status of questionnaire respondents of agrarian communities in Ibaji and Igalamela-Odolu Local Government Areas, Kogi State (n = 295).

Characteristics	Frequency (%)

**House floor type**	
Mud	149 (50.5)
Cement	138 (46.8)
Tiles	8 (2.7)
**Electricity and Appliances**	
Electricity	123 (41.7)
Radio	170 (57.6)
Television	78 (26.4)
Refrigerator	21 (7.1)
**Source of Water**	
Tap	153 (51.9)
River	135 (45.8)
Well	15 (5.1)
Rain	11 (3.7)
Toilet facility possession	128 (43.4)
*Types of Toilet*	
Pit	84 (28.5)
Water closet	32 (10.8)
Bucket	6 (2.0)
Others	6 (2.0)
**Wipe Used after Toilet**	
Toilet paper	28 (9.5)
Water	172 (58.3)
Plant leaf	95 (32.2)

n = number examined.

### Knowledge, perception, attitude and practices

Summary of knowledge, attitude and practices of the questionnaire respondent are presented in Table 4. Almost half (48.8%) of the respondents think they could get infected by worms but only 8.8% know how they could get infected; and only 16.6% try to prevent infection. A high proportion of respondents defecate in the open (59.3%). Though 64.7% eat food with bare hands (without cutleries), 88.5% washed their hands after using the toilet.

**Table 4:** Knowledge, perception, attitude and practice of questionnaire respondents in agrarian communities of Ibaji and Igalamela-Odolu Local Government Area, Kogi State (n = 295).

Characteristics	Frequency (%)

**Knowledge and Perception**	
Know parasitic worm	115 (39.0)
Think could get worms	144 (48.8)
Know how to get worms	26 (8.8)
Ever had worm infection	109 (36.9)
**Attitude and Practices**	
Try to prevent worm infection	49 (16.6)
***Hygiene***	
Defecate in open	175 (59.3)
*Wipe after toilet*	
Toilet paper	28 (9.5)
Water	172 (58.3)
Leaf	95 (32.2)
Wash hand after toilet	261 (88.5)
Wash hand with soap	169 (57.3)
Eat meal with bare hands (without cutleries)	191 (64.7)
***Home/farm activities***	
Eat in the farm	265 (86.8)
Wear shoe to farm	272 (92.2)
Wear shoe while working in farm	170 (57.6)
Wear shoe at home	219 (74.2)
Child/ward play in sand	276 (93.6)
Have animal(s) at home	209 (70.8)
Types*	
Dog	19 (6.4)
Pig	6 (2.0)
Goat	146 (49.5)
Chicken	162 (54.9)
Cow	5 (1.7)
Others	2 (0.7)
***Use of health services***	
*have medical centre near home	262 (88.8)
*been sick one or more times in past year	242 (82.0)
Visited the doctor one or more times in the past year	184 (62.4)
*have received medicine for worm	93 (31.5)
*When received medicine for worm?	
None	203 (68.8)
Months ago	40 (13.6)
Years ago	53 (18.0)

* Included in the section for convenience. n = number examined.

### Socioeconomic Status (SES) in Ibaji and Igalamela-Odolu LGAs

**Figure 3:**
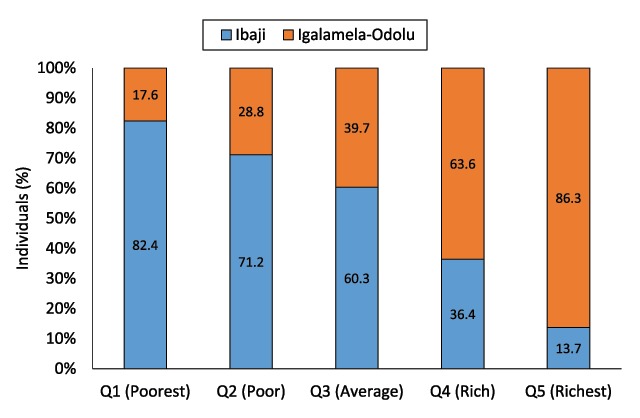
Distribution of wealth among individuals in Ibaji and Igalamela-Odolu Local Government Areas.

A total of 68 (23.1%) of the respondents were classified as poorest based on the 20-assets criteria. Those in richest category were 73 (24.7%) while poor, average and rich were slightly fewer (Table 5). Homes with mud floor was mainly associated with poverty, 68 (45.6%) and 49 (32.9%) of those that used mud floor were poorest and poor respectively. Majority of the people who had cement floors at home where in the fourth and fifth quintiles. There were very high significant differences in the distribution of floor types by wealth quintile (p < 0.0001). Electricity and electrical appliances such as radio, television and refrigerators were common in homes of the rich and richest members of the communities. The poorest and poor were relatively more likely to practice open defecation and constituted 39.4% and 25.1% respectively of 175 who practiced it. This contrasts strongly with 12.0% and 6.3% of the rich and richest people respectively. Majority of people in Igalamela-Odolu LGA had better wealth status than those in Ibaji LGA (Figure 3).

**Table 5:** Wealth quintiles and relative possession of assets among agrarian communities in Ibaji and Igalamela-Odolu Local Government Areas, Kogi State.

Descriptors	Wealth Quintiles (%)					Total	P**

	Q_1_ (Poorest)	Q_2_ (Poor)	Q_3_ (Average)	Q_4_ (Rich)	Q_5_ (Richest)		

**Total**	**68 (23.1)**	**52 (17.6)**	**58 (19.7)**	**44 (14.9)**	**73 (24.7)**	**295**	
*Floor type*							
Mud	68 (45.6)	49 (32.9)	30 (20.1)	2 (1.3)	0 (0.0)	149	<0.0001
Cement	0 (0.0)	3 (2.1)	28 (19.2)	42 (28.8)	73 (50.0)	146	<0.0001
*Electricity & Appliances*							
Electricity	2 (1.6)	13 (10.6)	17 (13.8)	28 (22.8)	63 (51.2)	123	<0.0001
Radio	9 (5.3)	28 (16.5)	34 (20.0)	29 (17.1)	70 (41.2)	170	<0.0001
Television	10 (12.8)	6 (7.7)	14 (17.9)	17 (21.8)	31 (39.7)	78	<0.0001
Refrigerators	1 (4.8)	0 (0.0)	4 (19.0)	6 (28.6)	10 (47.6)	21	0.005
*Source of water*							
Pipe-borne	3 (2.0)	16 (10.5)	37 (24.2)	27 (17.6)	70 (45.8)	153	<0.0001
River	67 (49.6)	35 (25.9)	18 (13.3)	15 (11.1)	0 (0.0)	135	<0.0001
Well	1 (6.7)	3 (20.0)	3 (20.0)	6 (40.0)	2 (13.3)	15	0.052
Rain	0 (0.0)	0 (0.0)	0 (0.0)	7 (63.6)	4 (36.4)	11	<0.0001
Have toilet	2 (1.6)	10 (7.8)	24 (18.8)	28 (21.9)	64 (50.0)	128	<0.0001
*Toilet types*							
Pit	0 (0.0)	5 (6.0)	12 (14.3)	17 (20.2)	50 (59.5)	84	<0.0001
Water closet	1 (3.1)	0 (0.0)	10 (31.2)	7 (21.9)	14 (43.8)	32	<0.0001
Bucket	0 (0.0)	1 (16.7)	1 (16.7)	4 (66.7)	0 (0.0)	6	0.007
Others*	1 (16.7)	3 (50.0)	2 (33.3)	0 (0.0)	0 (0.0)	6	0.149
*Wipes*							
Toilet paper	9 (32.1)	4 (14.3)	3 (10.7)	1 (3.6)	11 (39.3)	28	0.096
Water	23 (13.4)	28 (16.3)	40 (23.3)	27 (15.7)	54 (31.4)	172	<0.0001
Plant leaf	36 (40.0)	20 (22.2)	14 (15.6)	14 (15.6)	6 (6.7)	90	<0.0001
*Possess animals*							
Chicken	30 (18.5)	27 (16.7)	32 (19.8)	26 (16.0)	47 (29.0)	162	0.175
Goat	38 (26.0)	16 (11.0)	37 (25.3)	25 (17.1)	30 (20.5)	146	0.003
Defecate in open^†^	61 (34.9)	44 (25.1)	38 (21.7)	21 (12.0)	11 (6.3)	175	<0.0001

Q^1^ to Q^5^ are first to fifth socio-economic status quintiles; values are presented as number of respondent possessing the asset (%). * other unspecified toilet types. ^†^ Open defecation was not included as asset in the PCA but was include here for ease of presentation. ** p-value from chi-square comparison of possession of asset between quintiles.

A summary of how STH infection was distributed in relation to the 20 assets is provided in supplementary file 2. Four assets were each significant determinant of STH infection. The single most important determinant was the possession of a toilet (χ^2^ = 13.469, p < 0.0001), especially pit toilet (χ^2^ = 10.222, p = 0.0001). The other two were ‘having water to wash after toilet’ (χ^2^ = 3.854, p = 0.050) and ‘using plant leaf as toilet paper’ (χ^2^ = 4.759, p = 0.029), and both more likely are associated with use of toilet and open defecation respectively. As was expected, people who had cement floor, electricity, television and pipe-borne water had lower cases of infection compared to those who did not (S2 Figure). Surprisingly, refrigerator as an indicator of wealth was associated with greater infection.

### Risk for infection with soil-transmitted helminths in Ibaji and Igalamela-Odolu LGAs

Risks of STH infection due to SES, knowledge, attitude and practices is presented in Table 6. Risk of STH infection was similar between the first four wealth quintiles: poorest, poor, average and rich. However, risk of infection was significantly lower by 60% in the richest wealth quintile compared to the poorest quintile (PR = 0.4843, 95% CI = 0.2704–0.8678, p = 0.015). People that claimed to know parasitic worms, that think they could get worms, know how to get worms, and believed they had previously been infected by worms had little to no reduction in risk of STH infection when compared to those whose knowledge and perceptions were otherwise (Table 6). Open defecation was one major factor associated with significant increase in STH risk. People who practiced open defecation were two times more likely to harbor STH compared to those who did not (PR = 1.7878, 95% CI = 1.2366–2.5846, p = 0.00201). Compared to non-possession of toilet facilities, pit latrine and water closet toilet each reduced STH infection by approximately 50% (p < 0.05). Washing hands after toilet use, and wearing shoes while working in farms each also significantly lowered the risk of STH infections. Though washing of hands after toilet use significantly lowered the risk of infection, using soap for this purpose had no to little effect (Table 6).

**Table 6:** Risk of soil-transmitted helminths infection associated with socio-economic status (SES), knowledge, perception, attitude and practices among agrarian communities in Ibaji and Igalamela-Odolu Local Government Area, Kogi State (n = 295).

Determinants	PR (95% CI)	p

**SES**		
Q1 (Poorest)	1	
Q2 (Poor)	0.9938 (0.6182–1.5979)	0.97967
Q3 (Average)	1.2193 (0.7990–1.8608)	0.35789
Q4 (Rich)	1.1127 (0.6937–1.7849)	0.65773
Q5 (Richest)	0.4843 (0.2704–0.8678)	0.01483
**Knowledge and Perception**		
Know parasitic worms	0.7943 (0.5654–1.1159)	0.18425
Think could get worms	0.8095 (0.5876–1.1150)	0.19580
Know how to get worms	0.7705 (0.4006–1.4817)	0.43446
Ever had worm infection	0.9451 (0.6784–1.3166)	0.73850
**Attitude and Practices**		
Try to prevent worm infection	1.0160 (0.6666–1.5487)	0.94104
***Hygiene***		
Defecate in open	1.7878 (1.2366–2.5846)	0.00201
*Toilet types		
No toilet	1	
Pit	0.4694 (0.2967–0.7428)	0.00123
Water closet	0.5074 (0.2577–0.9991)	0.04968
Bucket	0.7731 (0.2460–2.4294)	0.65962
Others**	1.1597 (0.5113–2.6303)	0.72285
Wash hand after toilet	0.6437 (0.4404–0.9407)	0.02288
Wash hand with soap	0.9278 (0.6755–1.2744)	0.64360
Eat meal with bare hands (without cutleries)	1.2295 (0.8674–1.7429)	0.24578
***Home/farm activities***		
Eat in the farm	1.1299 (0.6855–1.8625)	0.63202
Wear shoe to farm	1.6235 (0.7354–3.5843)	0.23039
Wear shoe while working in farm	0.7209 (0.5264–0.9872)	0.04130
Wear shoe at home	0.9048 (0.6383–1.2825)	0.57396
***Use of health services***		
Have medical centre near home*	0.9342 (0.5769–1.5125)	0.78177
Been sick one or more times in past year*	1.0822 (0.7052–1.6606)	0.71780
Visited the doctor one or more times in the past year	0.8474 (0.6166–1.1646)	0.30751
Have received medicine for worm*	0.8331 (0.5813–1.1940)	0.32000
When received medicine for worm?*		
None	1	
Months ago	0.7544 (0.4418–1.2880)	0.30178
Years ago	0.8441 (0.5402–1.3189)	0.45664

N = number examined. PR = prevalence ratio; CI = confidence interval. * Included in the section for ease of result presentation. ** other toilet types apart from the ones listed. Except SES, types of toilet and “when medicine was received for worm” which has 1 indicated as reference, the reference value for all other determinants is 1 and represent the group that responded “no” to each of the questions concerned.

Risk of infection with either *A. lumbricoides*, hookworms or *T. trichiura* was lowest at the fifth wealth quintile, though it was generally not significant statistically. Those that perceived they could get infected by worms had 50% significantly lower risk of hookworm infection (PR = 0.4691, 95% CI = 0.2777–0.7925, p = 0.00467); though such perception had no significant impact on *A. lumbricoides* and *T. trichiura* infection. People who reported previous worm infections were over 200% more likely to be positive for *T. trichiura* (PR = 3.0716, 95% CI = 1.0564–8.9312, p = 0.03933), compared to those who did not. Those that tried to prevent worm infection had reduced risk of hookworm and *T. trichiura* infections though not significantly, however there was twofold increased risk of *A. lumbricoides* (PR = 2.1516, 95% CI = 1.2774–3.6241, p = 0.00397). Open defecation generally increased the risk of STH infection, but the impact was only significant for hookworms (PR = 2.0082, 95% CI = 1.1465–3.5173, p = 0.01476); the impact on *T. trichiura* was large but not significant (PR = 4.1143, 95% CI = 0.9377–18.0529, p = 0.06084). Possession of a pit latrine reduced the risk of all three STH, the effect was only significant for hookworms (PR = 0.3699, 95% CI = 0.1823–0.7506, p = 0.00587). Water closet toilets only significantly reduced the risk of hookworm infection by over 70% (PR = 0.2473, 95% CI = 0.0619–0.9518, p = 0.04227). Wearing of shoes while working in farm or while at home lowered the risk of infection with all three worms, but this was only significant for *A. lumbricoides* (PR = 0.4507, 95% CI = 0.2673–0.7598, p = 0.00278) and *T. trichiura* (PR = 0.3470, 95% CI = 0.1258–0.9573, p = 0.04091).

## Discussion

Overall prevalence of STH in Ibaji and Igalamela-Odolu LGAs was moderate (34.2%). This falls within the 16.2–39.0% range among LGAs of Kogi State as earlier reported by the Nigerian Federal Ministry of Health [18]. However, the FMOH survey was only school-based. Most STH prevalence surveys in Nigeria were carried out among school children: Sowemino and Asaolu obtained prevalence of 34.4% in Ile-Ife, Osun State; 26.66% was reported in Rivers State, 80.9% among Almajiri children in northern Nigeria, and 30.3% in Imo state [9,31,32,33]. Differences in prevalence obtained in various parts of the country relative to the present study may be attributable to environmental, ecological and anthropogenic factors that prevailed in each area. Moreover, methodological differences between studies could also be an issue; while floatation techniques were applied in most of these surveys, the standard Kato-Katz technique was employed in this study. Comparative studies on floatation and Kato-Katz techniques have reported both similar and disparate performance depending on the helminth of interest [34,35,36]. Mean intensity of STH was low; moderate to high intensity was below 1% in the study population. This finding holds a promise in terms of control of STH in the population.

An important aspect of the result is the similarity in infection among the four age groups (preschool, school, women of reproductive age and men of same age, and older at-risk groups). This further emphasizes need for an extension of preventive chemotherapy to other at-risk groups, especially in settings such as the one covered by this study where majority of the inhabitants are at risk. The finding corroborates STHAC recommendation [16,17]. Though women of reproductive age were considered along with men of similar age group (52.8% vs. 47.2%), there are no bases for expecting that helminth burden will be any different between men and women considering the locality in question. Infection was also not significantly different between male and female (unreported). Restricting mass drug administration (MDA) to preschool-aged and school-aged in agrarian communities of Ibaji and Igalamela-Odolu LGAs, and other localities with similar attributes only increases the risk of re-infection after MDA. Extending drugs to all age groups may, however, increase the risk for antihelminthic drug resistance which needs to be monitored.

Factors associated with STH infection were identified in this study. Socio-economic and demographic factors such as poverty, lack of portable water, occupation, age, and attitudes such as poor sanitation and poor sewage disposal have been recognized as determinants of STH distribution in endemic areas [15,37]. From the asset-based approach used in this study, the number of very poor and very rich households was almost equal in the study area. While the asset-based approach employed effectively classified the household into wealth quintiles, number and types of assets considered generally influence classifications [27]. From the present study, people at the fifth wealth quintile (richest) were less likely to harbour STH. Possession of assets indicative of good SES such as cement floor, electricity, radio, television, pipe-borne water and toilet facilities such as pit latrine and water closets reduced the risk of STH. Open defecation which was common among people in the first, second and third wealth quintile had a major risk for STH infection. It has been stated previously that improper sewage disposal is a major factor for STH infection [38]. Availability of toilet facilities in a household is a matter of social class proving the saying that poverty breeds diseases. Mud and hut house is associated with increased STH infection [39]. Mud has the tendency of retaining parasite eggs even when it has been apparently cleaned. Mud floor which was assigned strong negative factor score by statistical analysis is one of the strong indicators of poverty in the LGAs. In addition, cement floor alone was one of the SES assets that strongly reduced the risk of infection with STH. Thus, STH infection in the LGAs can largely be blamed on SES. Though it has been suggested by Becker et al., that it is unlikely that significant impact can be made between 2018 and 2020 concerning socio-economic status of people in STH endemic areas [17]. This therefore suggests, that other approaches such as preventive chemotherapy are more important if the WHO 2020 target is to be achieved. The authors share the same view, but would wish to emphasize that in order to sustain gains, a sustainable approach against STH requires improved SES.

Knowledge, attitude and practices were other strong determinants of STH infections in the LGAs. Fifty-nine percent of the respondents admitted defecating in the open mainly due to lack of toilets. Risk of STH infection was generally higher among those who practiced open defecation. This was especially obvious for hookworm and *T. trichiuria*. This corroborates reports from Nigeria and other parts of the world [39,40]. Regular visits to defecation spots increase chances of a hookworm infection because the infective stages of these parasites develop around defecation sites where the eggs were released and are always ready to strike and infect an unsuspecting host. Open defecation can be reduced through health education and by improving the SES of communities affected through provision of amenities such as pit latrines and water closet toilets.

Hand washing after toilet use lowered the risk of acquiring STH. Although auto-infection from freshly passed stool is not possible since period of incubation in the soil is required by STH, habitual hand washing after toilet use is an indication of high sense of personal hygiene. More so, individuals, especially children, who use regular open defecation sites are more likely to soil their hands with previously contaminated soil. Therefore hand washing protects against STH. It has been advocated that health education in the aspect of hand washing with soap be intensified among communities where STH infection occurs [41]. Though the present study found no special role played by soap. The importance of water, sanitation and hygiene (WASH) which was re-affirmed by STHAC is emphasized by the present study.

Chances of having STH infection were also lowered significantly by wearing shoes while working on the farm. This act provided some level of protection against the three STH, and the effect was significant against *A. lumbricoides*. Not wearing of shoes was similarly reported as a risk factor for hookworm infection in northwestern Ethiopia [42]. The infective hookworm larvae from soil enter into the host by active penetration of unbroken skin especially the feet that are often in contact with the soil. Shoe wearing therefore, reduces the risk of hookworm infection. Though unrelated to *A. lumbricoides* infection, shoe wearing prevents contamination of feet which could indirectly lead to infection when individuals touch such contaminated feet and eat with unwashed hands afterwards.

## Conclusion and recommendations

In conclusion, this study reports a moderate prevalence and light intensity of STH infection in the study population indicating its endemicity in the study area and therefore a public health problem that requires attention. A number of risk factors associated with STH transmission was identified. These are mainly socio-economic, attitude and practices. Based on the findings, it is hereby recommended that

1. Mass preventive chemotherapy against STH infection through the interventions of the Ministry of Health and other health organizations should be extended to all age groups in the affected communities. This is especially important to prevent risk of re-infection by other at-risk adults.
2. Health education and awareness on transmission and health impact of STH should be emphasized. The study further affirms water, sanitation and hygiene (WASH) as integral STH control strategy.
3. Effort should be made at improving SES in places where STH has moderate to high endemicity such as Ibaji and Igalamela-Odolu LGAs through provision of basic amenities such as pipe-borne water, electricity, good housing and especially pit latrines and water closet toilets.

## Additional Files

The additional files for this article can be found as follows:

10.5334/agh.2563.s1 S1 Table. Supporting information from the asset-based socio-economic status.

10.5334/agh.2563.s2 S2 Figure. **Proportion of people positive and negative for soil-transmitted helminthiasis who had different assets.** The five key determinants are highlighted in dark-walled rectangles. Possession of toilet was the most important determinant, usage of plant leaf or water is closely associated with possession of toilet (open defecation is not an asset, but was included here for convenience of presentation).
