# Supplementary material for: Risk of Soil-Transmitted Helminthiasis among Agrarian Communities of Kogi State, Nigeria
Source: Ann Glob Health. 2019 Sep 11;85(1):120. doi: 10.5334/aogh.2563 (PMC6743035; doi:10.5334/aogh.2563)
Supplement: S1 Table. — Supporting information from the asset-based socio-economic status. [file agh-85-1-2563-s1.pdf]

S1 Table. Supporting information from the asset-based socio-economic status

Component Matrix[a]

	Component						
	1	2	3	4	5	6	7
have toilet	.808			.163	.203		.159
cement	.804			-.189	-.304		
mud	-.804			.189	.304		
electricity	.652	.311		.226	-.178		
river	-.646	.461		.118	.263		.184
have tap water	.617	-.510		-.126	-.181		-.166
leaf	-.577	.358		-.131	-.435	-.368	.170
Pit	.561	-.419		.182			.486
radio	.517	.267		.254	-.199	-.123	.177
refrigerator	.377	.600	-.116		.238		-.180
television	.433	.588		.277	.131		
rain	.192	.568	-.125	-.279		-.255	-.114
Watercloset	.398	.425	-.251	-.370	.248	.110	-.371
Bucket	.203	.118	.783	.190			-.255
well		.163	.758	.157	-.233		-.201
Others		.289	-.173	.650	.144	-.163	.103
chicken	.159		.415	-.475	.102		.459
water	.519	-.499			.526	-.198	-.231
goat	.130	.177	.345	-.326	.479		.389
toiletpaper		.173	-.102		-.194	.935	

Extraction Method: Principal Component Analysis.

a. 7 components extracted.

Component Score Coefficient Matrix

	Component						
	1	2	3	4	5	6	7
mud	-.253	-.066	.016	.066	.078	.002	-.019
cement	.253	.066	-.016	-.066	-.078	-.002	.019
electricity	.168	.019	.095	.207	-.058	.002	-.014
radio	.167	-.039	.020	.240	-.094	-.012	-.002
television	.017	.131	.098	.299	.014	.101	.053
refrigerator	-.026	.329	.015	.083	.049	.058	.031
have tap water	.166	-.041	-.007	-.204	.119	-.049	-.131
river	-.184	.050	-.087	.218	-.087	.032	.126
well	.014	-.040	.526	-.033	-.049	.042	-.017
rain	.059	.292	-.027	-.016	-.155	-.173	.036
have toilet	.094	-.004	-.044	.184	.172	.069	.123
Pit	.148	-.289	-.167	.159	.096	.077	.192
Watercloset	-.028	.432	-.058	-.141	.100	.047	-.031
Bucket	-.034	-.001	.529	.002	.063	-.040	.014
Others	-.041	-.061	-.028	.470	.023	-.065	-.123
toiletpaper	-.009	-.018	.004	-.056	.021	.787	-.019
water	-.065	.029	.010	.000	.387	-.271	-.008
leaf	.067	-.030	-.013	.005	-.412	-.209	.013
goat	-.088	.033	-.007	.008	.070	.022	.560
chicken	.047	-.052	.021	-.116	-.097	-.037	.548

Extraction Method: Principal Component Analysis.

Rotation Method: Oblimin with Kaiser Normalization.

Component Scores.

Statistics		
REGR factor score 1 for analysis 1		
N	Valid	295
	Missing	0
Mean		0E-7
Std. Error of Mean		.05822225
Median		-.0641089
Std. Deviation		1.00000000
Variance		1.000
Minimum		-1.61857
Maximum		1.71463
Percentiles	20	-1.0724367
	25	-.9104205
	40	-.4875375
	50	-.0641089
	60	.3660927
	75	.9864561
	80	1.0723084

REGR factor score 1 for analysis 1					
		Frequency	Percent	Valid Percent	Cumulative Percent
Valid	-1.61857	3	1.0	1.0	1.0
	-1.52452	2	.7	.7	1.7
	-1.51844	2	.7	.7	2.4
	-1.51818	1	.3	.3	2.7
	-1.47948	1	.3	.3	3.1
	-1.44246	5	1.7	1.7	4.7
	-1.42440	1	.3	.3	5.1
	-1.34842	1	.3	.3	5.4
	-1.34258	3	1.0	1.0	6.4
	-1.34234	3	1.0	1.0	7.5
	-1.30362	6	2.0	2.0	9.5
	-1.24854	12	4.1	4.1	13.6
	-1.24830	1	.3	.3	13.9
	-1.18684	1	.3	.3	14.2
	-1.18345	1	.3	.3	14.6
	-1.16648	4	1.4	1.4	15.9
	-1.15632	1	.3	.3	16.3
	-1.14768	1	.3	.3	16.6
	-1.11126	1	.3	.3	16.9
	-1.10595	1	.3	.3	17.3
	-1.10459	5	1.7	1.7	19.0
	-1.07244	7	2.4	2.4	21.4
	-1.03347	2	.7	.7	22.0
	-1.01138	2	.7	.7	22.7
	-1.01054	1	.3	.3	23.1
	-1.00471	1	.3	.3	23.4
	-.97635	1	.3	.3	23.7
	-.96619	1	.3	.3	24.1
	-.92459	1	.3	.3	24.4
	-.91042	2	.7	.7	25.1
	-.90652	1	.3	.3	25.4
	-.84558	3	1.0	1.0	26.4
	-.82861	2	.7	.7	27.1
	-.82738	1	.3	.3	27.5
	-.82511	2	.7	.7	28.1
	-.81993	1	.3	.3	28.5
	-.81864	1	.3	.3	28.8
	-.76352	2	.7	.7	29.5
	-.74305	9	3.1	3.1	32.5

	-.73456	7	2.4	2.4	34.9
	-.67544	1	.3	.3	35.3
	-.66947	1	.3	.3	35.6
	-.64317	1	.3	.3	35.9
	-.64278	2	.7	.7	36.6
	-.60697	1	.3	.3	36.9
	-.58671	1	.3	.3	37.3
	-.58128	1	.3	.3	37.6
	-.57946	1	.3	.3	38.0
	-.55140	2	.7	.7	38.6
	-.52728	1	.3	.3	39.0
	-.51292	2	.7	.7	39.7
	-.48754	3	1.0	1.0	40.7
	-.48724	2	.7	.7	41.4
	-.46707	1	.3	.3	41.7
	-.44857	1	.3	.3	42.0
	-.44827	1	.3	.3	42.4
	-.43086	1	.3	.3	42.7
	-.40518	2	.7	.7	43.4
	-.40128	1	.3	.3	43.7
	-.38711	1	.3	.3	44.1
	-.37303	1	.3	.3	44.4
	-.33682	1	.3	.3	44.7
	-.30723	3	1.0	1.0	45.8
	-.30530	3	1.0	1.0	46.8
	-.28873	1	.3	.3	47.1
	-.23221	1	.3	.3	47.5
	-.22518	1	.3	.3	47.8
	-.17505	1	.3	.3	48.1
	-.14617	2	.7	.7	48.8
	-.12920	1	.3	.3	49.2
	-.08979	1	.3	.3	49.5
	-.07743	1	.3	.3	49.8
	-.06411	1	.3	.3	50.2
	-.06084	1	.3	.3	50.5
	-.05788	1	.3	.3	50.8
	-.03596	1	.3	.3	51.2
	-.03570	1	.3	.3	51.5
	.03064	1	.3	.3	51.9
	.03384	1	.3	.3	52.2
	.03648	1	.3	.3	52.5
	.03845	1	.3	.3	52.9

	.10094	1	.3	.3	53.2
	.11270	1	.3	.3	53.6
	.11913	1	.3	.3	53.9
	.14243	1	.3	.3	54.2
	.18649	2	.7	.7	54.9
	.19283	2	.7	.7	55.6
	.21133	2	.7	.7	56.3
	.22196	1	.3	.3	56.6
	.26615	1	.3	.3	56.9
	.27767	1	.3	.3	57.3
	.28687	3	1.0	1.0	58.3
	.30537	1	.3	.3	58.6
	.30592	1	.3	.3	59.0
	.36259	3	1.0	1.0	60.0
	.36843	1	.3	.3	60.3
	.37171	2	.7	.7	61.0
	.42808	1	.3	.3	61.4
	.44201	1	.3	.3	61.7
	.47233	1	.3	.3	62.0
	.48147	1	.3	.3	62.4
	.52406	1	.3	.3	62.7
	.54453	3	1.0	1.0	63.7
	.59568	1	.3	.3	64.1
	.61032	3	1.0	1.0	65.1
	.62040	1	.3	.3	65.4
	.62882	1	.3	.3	65.8
	.64651	1	.3	.3	66.1
	.64977	1	.3	.3	66.4
	.70437	1	.3	.3	66.8
	.72894	1	.3	.3	67.1
	.78642	2	.7	.7	67.8
	.79165	3	1.0	1.0	68.8
	.79374	2	.7	.7	69.5
	.82539	1	.3	.3	69.8
	.86543	1	.3	.3	70.2
	.87177	1	.3	.3	70.5
	.88047	3	1.0	1.0	71.5
	.89828	1	.3	.3	71.9
	.90231	1	.3	.3	72.2
	.91398	4	1.4	1.4	73.6
	.94749	2	.7	.7	74.2
	.97645	2	.7	.7	74.9

	.98646	1	.3	.3	75.3
	1.00244	3	1.0	1.0	76.3
	1.00478	1	.3	.3	76.6
	1.04154	3	1.0	1.0	77.6
	1.04224	4	1.4	1.4	79.0
	1.04762	1	.3	.3	79.3
	1.04787	2	.7	.7	80.0
	1.07842	1	.3	.3	80.3
	1.08121	1	.3	.3	80.7
	1.08450	1	.3	.3	81.0
	1.12103	1	.3	.3	81.4
	1.12430	1	.3	.3	81.7
	1.12749	2	.7	.7	82.4
	1.14142	2	.7	.7	83.1
	1.14192	3	1.0	1.0	84.1
	1.15645	1	.3	.3	84.4
	1.16326	1	.3	.3	84.7
	1.18696	1	.3	.3	85.1
	1.18931	1	.3	.3	85.4
	1.19290	1	.3	.3	85.8
	1.21834	2	.7	.7	86.4
	1.22347	2	.7	.7	87.1
	1.31981	1	.3	.3	87.5
	1.38331	10	3.4	3.4	90.8
	1.38954	1	.3	.3	91.2
	1.39118	1	.3	.3	91.5
	1.40187	1	.3	.3	91.9
	1.42228	4	1.4	1.4	93.2
	1.46537	2	.7	.7	93.9
	1.50433	5	1.7	1.7	95.6
	1.52240	1	.3	.3	95.9
	1.55941	4	1.4	1.4	97.3
	1.56549	2	.7	.7	98.0
	1.59838	3	1.0	1.0	99.0
	1.60446	2	.7	.7	99.7
	1.71463	1	.3	.3	100.0
	Total	295	100.0	100.0	

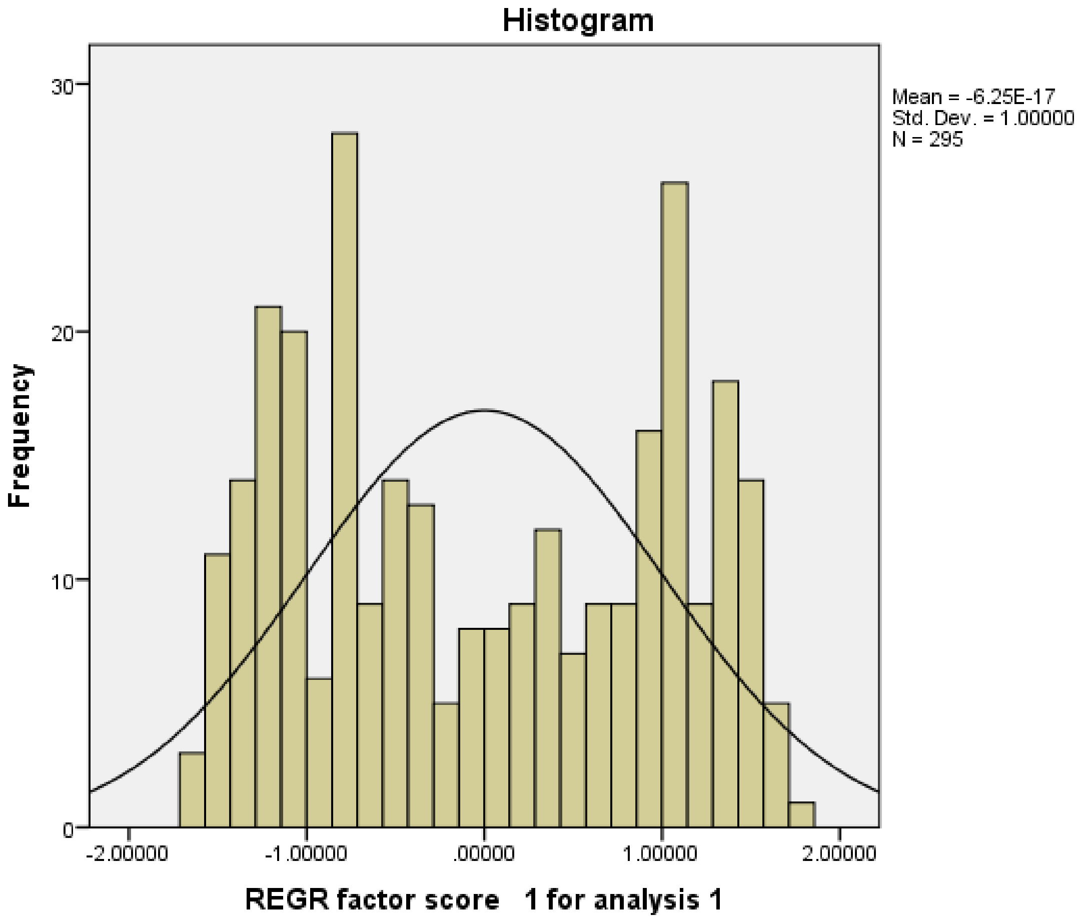
Histogram
Mean = -6.25E-17
Std. Dev. = 1.00000
N = 295
Frequency
30
20
10
0
-2.00000
-1.00000
.00000
1.00000
2.00000
REGR factor score 1 for analysis 1
