# Supplementary material for: Risk of Soil-Transmitted Helminthiasis among Agrarian Communities of Kogi State, Nigeria
Source: Ann Glob Health. 2019 Sep 11;85(1):120. doi: 10.5334/aogh.2563 (PMC6743035; doi:10.5334/aogh.2563)
Supplement: S2 Figure. — Proportion of people positive and negative for soil-transmitted helminthiasis who had different assets. The five key determinants are highlighted in dark-walled rectangles. Possession of toilet was the most important determinant, usage of plant leaf or water is closely associated with possession of toilet (open defecation is not an asset, but was included here for convenience of presentation). [file agh-85-1-2563-s2.pdf]

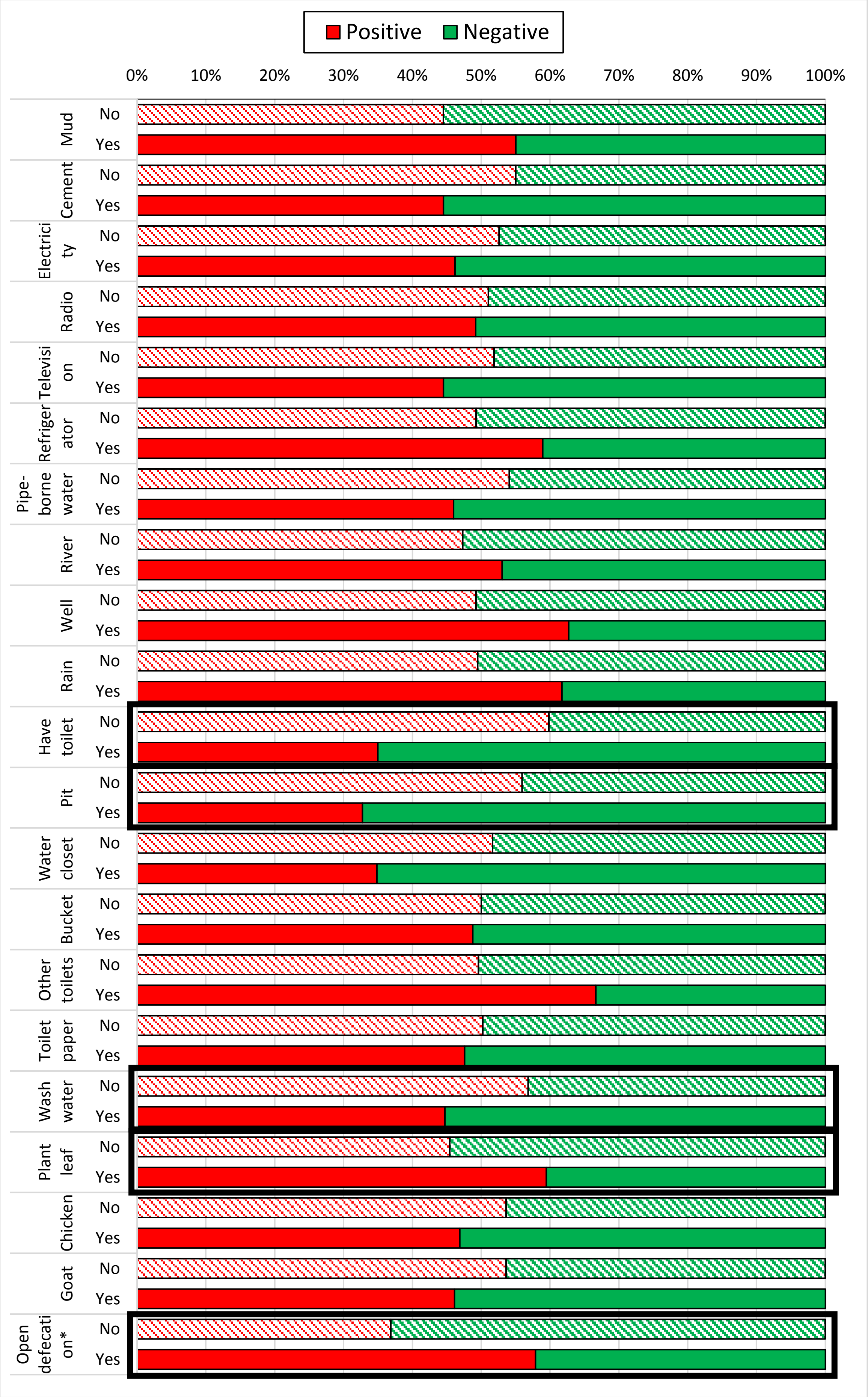

S2 Figure: Proportion of people positive and negative for soil-transmitted helminthiasis who had different assets. The five key determinants are highlighted in dark-walled rectangles. Possession of toilet was the most important determinant, usage of plant leaf or water is closely associated with possession of toilet (open defecation is not an asset, but was included here for convenience of presentation).
